# Supplementary material for: Modelling Skylarks (Alauda arvensis) to Predict Impacts of Changes in Land Management and Policy: Development and Testing of an Agent-Based Model
Source: PLoS One. 2013 Jun 6;8(6):e65803. doi: 10.1371/journal.pone.0065803 (PMC3675089; doi:10.1371/journal.pone.0065803)
Supplement: Supporting Information S4 — The skylark ODdox as a zipped archive. (ZIP) [file pone.0065803.s004.zip › Skylark_ODdox/structtpct.html]

ALMaSS Skylark ODdox: tpct Struct Reference


ALMaSS Skylark ODdox
 2.0


- Main Page
- Related Pages
- Classes
- Files

- Class List
- Class Index
- Class Hierarchy
- Class Members

Public Attributes

tpct Struct Reference

Used for storing farmers field size vectors.
More...

`#include <farm.h>`

List of all members.

|  |  |
| --- | --- |
| Public Attributes | |
| int | index |
| int | pct |

---

## Detailed Description

Used for storing farmers field size vectors.

---

## Member Data Documentation

int tpct::index

Referenced by UserDefinedFarm::AssignPermanentCrop().

int tpct::pct

Referenced by UserDefinedFarm::AssignPermanentCrop(), and CompPcts().

---

The documentation for this struct was generated from the following file:

- farm.h


- tpct
- Generated on Thu Jan 10 2013 13:15:38 for ALMaSS Skylark ODdox by
   1.8.1.1
